# Improvement of the intestinal epithelial barrier during laxative effects of phlorotannin in loperamide-induced constipation of SD rats

**DOI:** 10.1186/s42826-022-00152-1

**Published:** 2023-01-03

**Authors:** Ji Eun Kim, Hee Jin Song, Yun Ju Choi, You Jeong Jin, Yu Jeong Roh, Ayun Seol, So Hae Park, Ju Min Park, Hyun Gu Kang, Dae Youn Hwang

**Affiliations:** 1 Department of Biomaterials Science (BK21 FOUR Program), College of Natural Resources and Life Science/Life and Industry Convergence Research Institute/Laboratory Animal Resources Center, Pusan National University, Miryang, 50463 Korea; 2 Department of Food Science and Nutrition, College of Human Ecology, Pusan National University, Busan, 46241 Korea; 3 Veterinary Medical Center, Department of Veterinary Theriogenology, College of Veterinary Medicine, Chungbuk National University, Cheongju, 28644 Korea

**Keywords:** Intestinal epithelial barrier, Constipation, Phlorotannins, Laxative effects, Tight junction, Adherens junction

## Abstract

**Background:**

Disruptions of the intestinal epithelial barrier (IEB) are frequently observed in various digestive diseases, including irritable bowel syndrome (IBS) and inflammatory bowel disease (IBD). This study assessed the improvement in the IEB during the laxative activity of phlorotannin (Pt) harvested from *Ecklonia cava* in constipation by examining the changes in the expression of the regulatory proteins for the tight junction (TJ) and adherens junction (AJ), and inflammatory cytokines in Sprague Dawley (SD) rats with loperamide (Lm)-induced constipation after a Pt treatment.

**Results:**

The Pt treatment induced laxative activity, including the improvement of feces-related parameters, gastrointestinal transit rate, and histological structure of the mid colon in Lm-treated SD rats. In addition, significant recovery effects were detected in the histology of IEB, including the mucus layer, epithelial cells, and lamina propria in the mid colon of Lm + Pt treated SD rats. The expression levels of E-cadherin and p120-catenin for AJ and the ZO-1, occludin, and Claudin-1 genes for TJ in epithelial cells were improved remarkably after the Pt treatment, but the rate of increase was different. Furthermore, the Pt treatment increased the expression level of several inflammatory cytokines, such as TNF-α, IL-6, IL-1β, IL-13, and IL-4 in Lm + Pt treated SD rats.

**Conclusions:**

These results provide the first evidence that the laxative activity of Pt in SD rats with Lm-induced constipation phenotypes involve improvements in the IEB.

**Supplementary Information:**

The online version contains supplementary material available at 10.1186/s42826-022-00152-1.

## Background

The intestinal epithelial barrier (IEB) is the main selective physical barrier between the lumen and tissue in the gastrointestinal (GI) tract [1]. It helps maintain homeostasis through physical barriers protecting the body from harmful contents, selective filter regulating fluids, nutrients, and water, and secretion function of mucin and immunoglobulins [2]. The integrity and permeability of IEB are regulated by three junctional complexes that involve adherent junctions (AJ), desmosomes, and tight junctions (TJ) [2]. TJ is the most apical junction that makes a seal between the adjacent epithelial cells [3–5]. This complex contains transmembrane proteins, including claudins, occludin, and tricellulin, as well as cytoplasmic plaque proteins, such as zona occludens [2]. AJ is a specific cytoplasmic face linked to the actin cytoskeleton that plays a major role in initiating cell–cell contacts [6–8]. AJ consists of a few transmembrane proteins (E-cadherin and nectins) and some intracellular components (p120-catenin, β-catenin, and α-catenin) [9].

Severe disruption of IEB has been detected in some digestive diseases, including inflammatory bowel disease (IBD) and irritable bowel syndrome (IBS). IBD patients show an enhancement of paracellular permeability and the tissue penetration of large molecules and microbial pathogens [10]. This response is associated with TJ abnormalities, including alteration and redistribution of critical regulatory proteins [11–13]. In addition, similar alterations in the dysfunction of the mucus barrier permeability were detected in several IBD model animals, including IL-10 knock out (KO) mice, colitis mice induced with a treatment with dextran sodium sulfate (DSS) or dinitrobenzene sulfonic acid (DNBS) [14, 15]. Furthermore, IBS patients and animal models showed an alteration of IEB. They exhibited increased paracellular permeability, histological alteration of the mucus layer, and changes in the TJ proteins [16–19]. On the other hand, molecular changes in the IEB after the laxative effect of phlorotannin (Pt) in the mid colon of Sprague Dawley (SD) rats with Lm-induced constipation model are poorly understood, despite Pt having the potential to treat constipation.

Therefore, this study investigated the involvement of IEB during the laxative effect of Pt in the SD rats with Lm-induced constipation phenotypes. These results from our study present scientific evidence that the laxative activity of Pt may be linked to the improvement of Lm-induced IEB abnormality in the mid colon of SD rats.

## Results

### Confirmation of laxative activity of Pt in SD rats with Lm-induced constipation

**Fig. 1:**
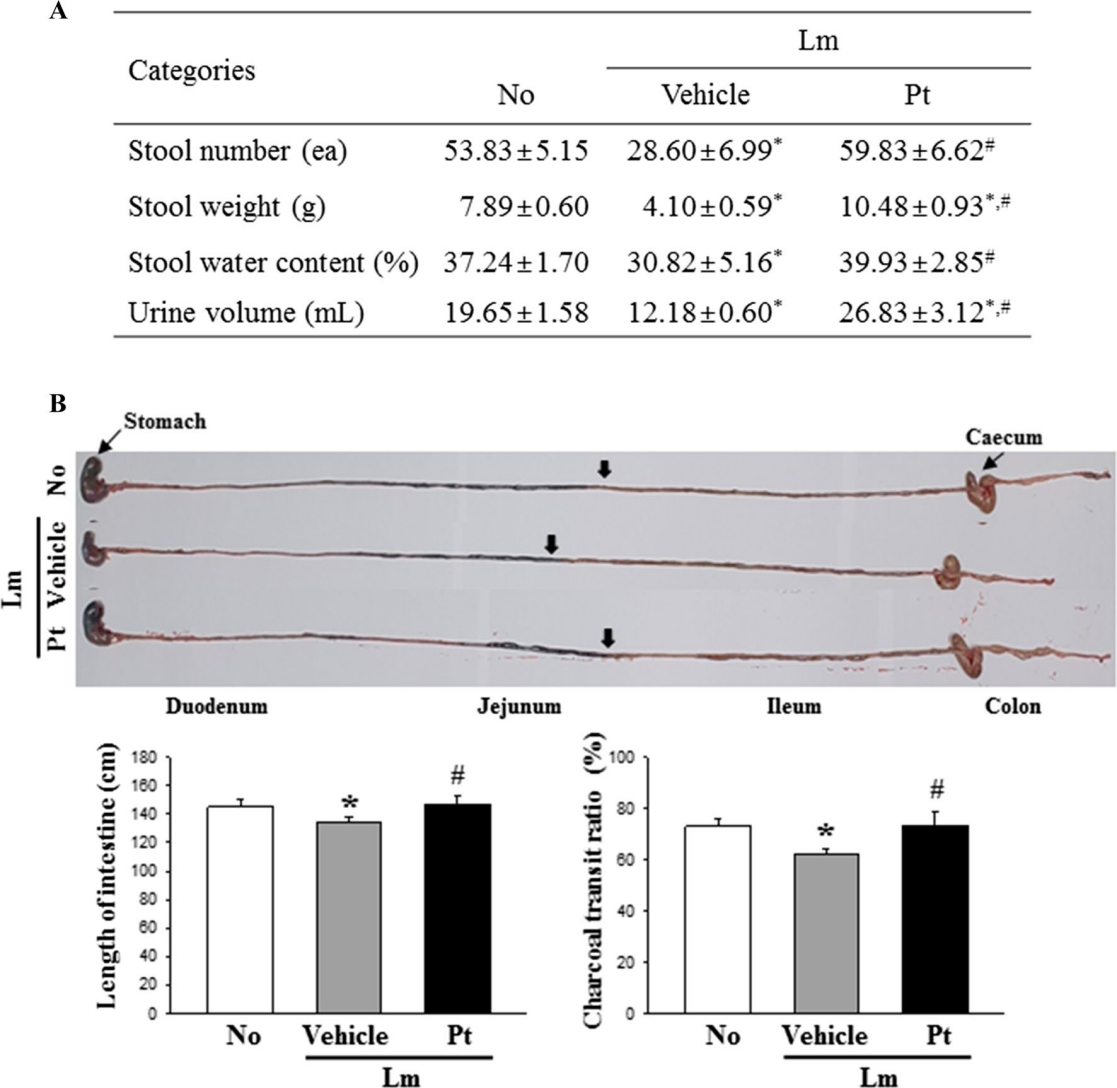
Feces-related parameters analyses and GI transit rate. **A** Feces-related parameters analyses. After collecting stools from the metabolic cage, their actual images were taken immediately, and sequentially the values of three parameters were analyzed. **B** GI transit rate and length. After measuring the distance of charcoal meal travel and total length of the GI tract, GI transit rate was then calculated based on these data. The feces collection and GI transit rate experiment were prepared from four to six rats per group, and parameters for these experiment was analyzed in duplicate. All values in results are represented as the means ± standard deviation (SD). *Indicated statically significance compared to the No treated SD rats, while # indicated statically significance compared to the Lm + Vehicle treated SD rats. Abbreviation: Lm, Loperamide; Pt, Phlorotannins

**Fig. 2:**
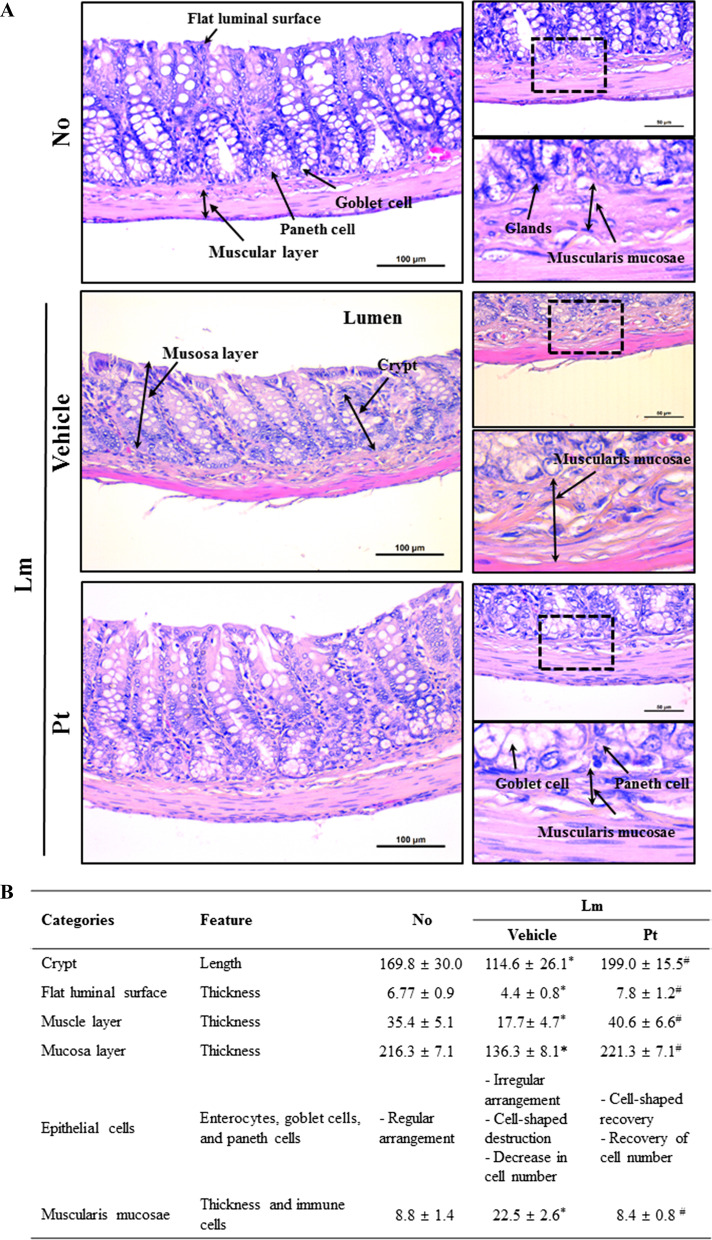
Histopathological structures of the mid colon in Lm + Pt treated SD rats. **A** Histopathological images. Alterations on the tissue sections stained with H&E solution were observed at 40× (left column) and 400× (right column) using an light microscope. **B** Value of histopathological parameters. Leica Application Suite was used to determine these values. The H&E stained tissue sections were prepared from four to six rats per group, and parameters for the histopathological parameters on the mid colon was analyzed in duplicate. All values in results are represented as the means ± standard deviation (SD). *Indicated statically significance compared to the No treated SD rats, while # indicated statically significance compared to the Lm + Vehicle treated SD rtas. Abbreviation: H&E, Hematoxylin and eosin; Lm, Loperamide

In a previous study, the Pt treatment induced laxative effects, including a decrease in the excretion-related parameters, recovery of colon histology, and inhibition of mucin secretion-related factors [20]. Therefore, the laxative activity of Pt in SD rats with Lm-induced constipation were first confirmed before examining the improvement of IEB in the mid colon. The level of three feces-related parameters, including total weight, total number, and their water contents, and urine volume, were enhanced significantly in the Lm + Pt cotreated rats compared to the Lm + Vehicle cotreated rats (Fig. 1A). In addition, similar alterations effects were remarkably detected in the level of GI transit rate and total length of colon. The levels of two factors were increased in SD rats with Lm-induced constipation phenotypes after the Pt administration (Fig. 1B). Furthermore, the changes in the stool parameters and GI transit rate were reflected entirely in the histology of the mid colon. The Pt treatment induced the recovery of the crypt length, the luminal surface thickness, and the muscle layer thickness (Fig. 2A, B). These results for laxative activity of Pt suggest that the Pt treatment may have laxative effects in SD rats with Lm-induced constipation phenotypes.

### Improvement in the histology of IEB during laxative effects of Pt

The changes in the mucosa layer, epithelial cells, and lamina propria in the histology of mid colon were analyzed in Lm + Pt treated SD rats after H&E staining to determine if the laxative activity of Pt are accompanied by changes in the histological structure of IEB. The average level of the mucosa layer thickness was lower in the Lm + Vehicle treated SD rats than in the No treated SD rats. On the other hand, above change was recovered significantly in the Lm + Pt treated SD rats compared to the Lm + Vehicle treated SD rats (Fig. 2A, B). In addition, remarkable changes in the epithelial cells, including enterocytes, paneth cells, and goblet cells, were detected. Destruction of the cell shape, decrease in the cell number, and an irregular arrangement of cells were observed after the Pt treatment (Fig. 2A, B). Furthermore, the Pt treatment induced a significant recovery of the thickness of the lamina propria, but the distribution of immune cells was not determined (Fig. 2A, B). These results suggest that the laxative activity of Pt are linked to an improvement of the histological structure of IEB in Lm treated SD rats with constipation phenotypes through structural regulation of the mucus layer, epithelial cells, and lamina propria.

### Improvement in the junctional complexes of IEB during laxative effects of Pt

**Fig. 3:**
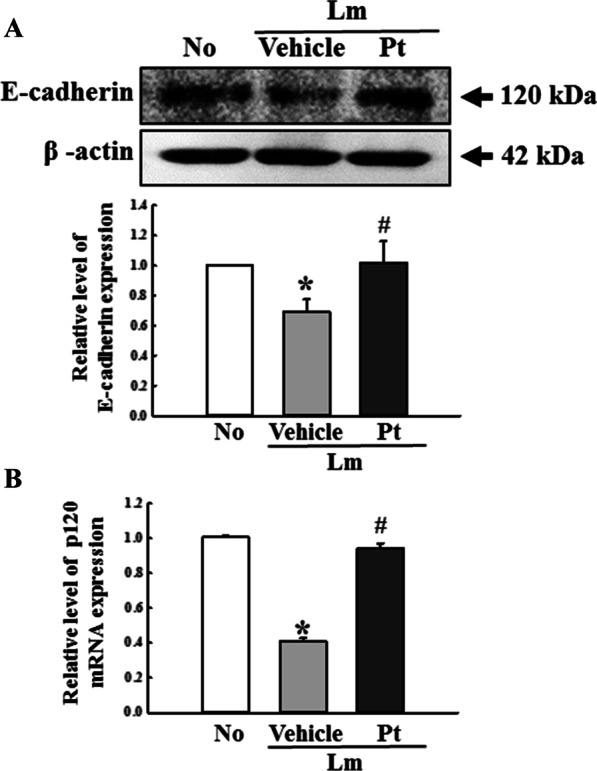
Expression of the AJ regulators in Lm + Pt treated SD rats. **A** Western blot of E-cadherin. After preparing the colon protein homogenates, the level of E-cadherin protein was measured by Western blotting analysis. **B** Level of p120-catenin mRNA. After preparing the total RNAs, the level of p120-catenin expression was determined by RT-qPCR analyses. The relative level of each protein band and mRNA was analyzed based on the level of β-actin. The total proteins homogenates and RNA extracts were prepared from four to six rats per group, and western blot and RT-qPCR analyses for each sample was analyzed in duplicate. All values in results are represented as the means ± standard deviation (SD). *Indicated statically significance compared to the No treated SD rats, while # indicated statically significance compared to the Lm + Vehicle treated SD rats. Abbreviation: Lm, Loperamide; Pt, Phlorotannins

**Fig. 4:**
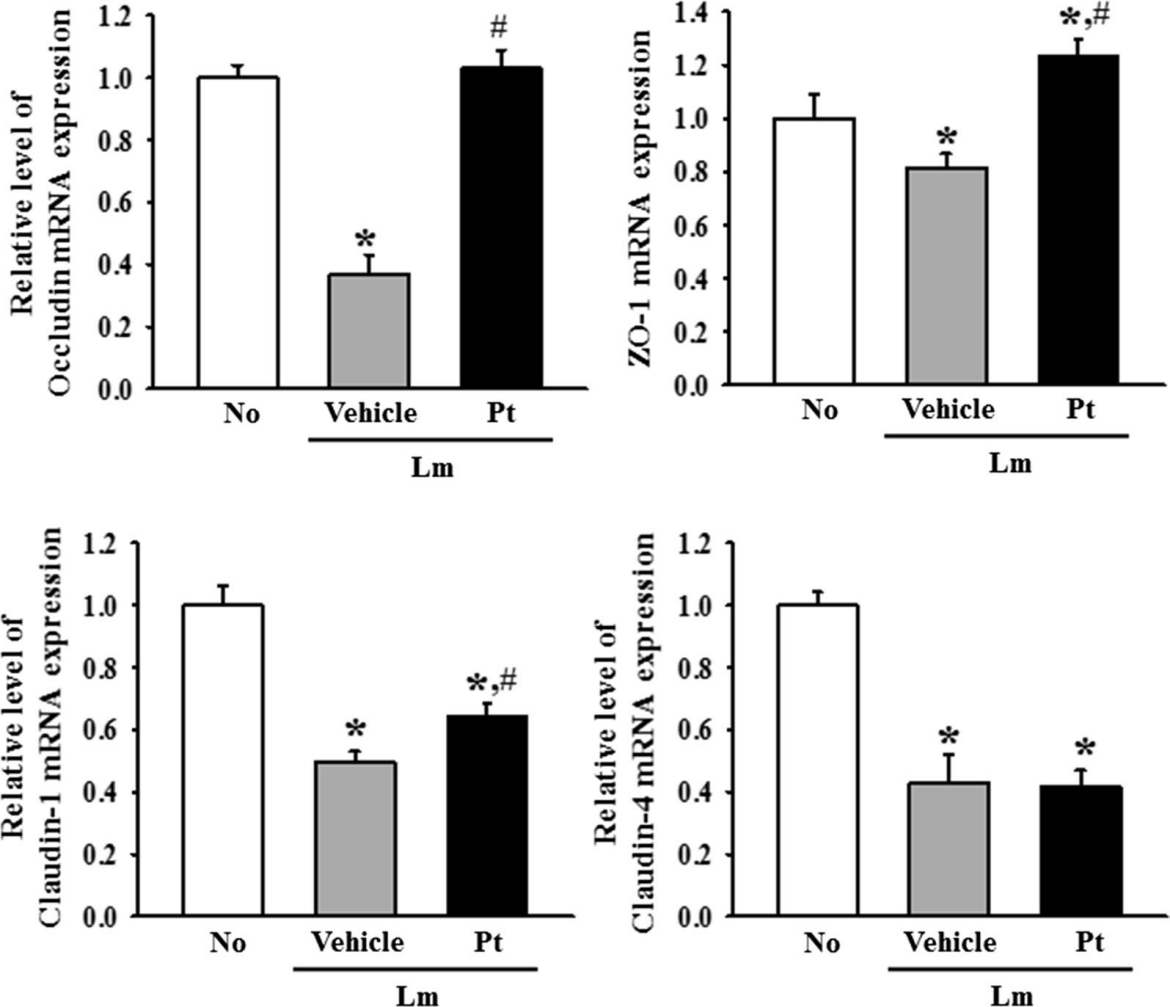
Expression of the TJ regulators in Lm + Pt treated SD rats. After the total RNA preparation, the transcript levels of occludin, ZO-1, claudin-1 and claudin-4 genes were determined by RT-qPCR analyses. The relative level of each transcript was determined based on the level of β-actin. The total RNA extracts were prepared from four to six rats per group, and RT-qPCR analyses for each sample was analyzed in duplicate. All values in results are represented as the means ± standard deviation (SD). *Indicated statically significance compared to the No treated SD rats, while # indicated statically significance compared to the Lm + Vehicle treated SD rats. Abbreviation: Lm, Loperamide; Pt, Phlorotannins

Alterations on the expression level of the key components for AJ and TJ were analyzed in mid colons of Lm + Pt treated SD rats with constipation phenotypes to determine if the Pt-induced improvements in the histological structure of IEB were accompanied by regulation of two junctional complexes between epithelial cells. First, the classical cadherin superfamily, including E-cadherin and the catenin family members, such as p120-catenin, were analyzed as the core of AJ. The levels of E-cadherins proteins and p120-catenin mRNA were significantly lower in the Lm + Vehicle treated SD rats than in the No treated SD rats. But, they were increased remarkably after the Pt administration (Fig. 3A, B). In addition, a similar response was detected in the mRNA levels of occludin, ZO-1, and claudin-1, even though the level of claudin-4 remained constant (Fig. 4). These results suggest that the laxative activity of Pt are linked to recovery of the junctional complex on IEB in SD rats with Lm-induced constipation phenotypes through the alternative regulation in the expression of AJ and TJ key components.

### Improvement in the inflammatory response of IEB during the laxative activity of Pt

**Fig. 5:**
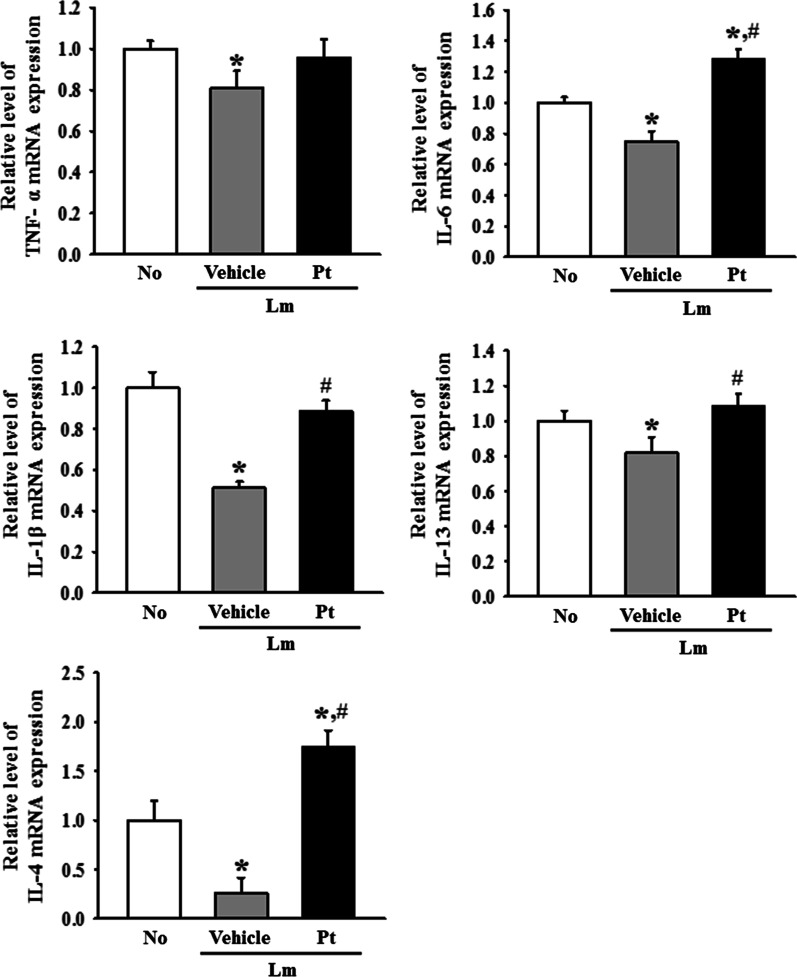
Expression of the inflammatory cytokines in Lm + Pt treated SD rats. The transcripts levels of TNF-α, IL-6, IL-1β, IL-13, and IL-4 genes were measured by RT-qPCR analyses. The mRNA level of each gene was determined based on the level of β-actin mRNA. The total RNA extracts were prepared from four to six rats per group, and RT-qPCR analyses for each sample was analyzed in duplicate. All values in results are represented as the means ± standard deviation (SD). *Indicated statically significance compared to the No treated SD rats, while # indicated statically significance compared to the Lm + Vehicle treated SD rats. Abbreviation: Lm, Loperamide; Pt, Phlorotannins

**Fig. 6:**
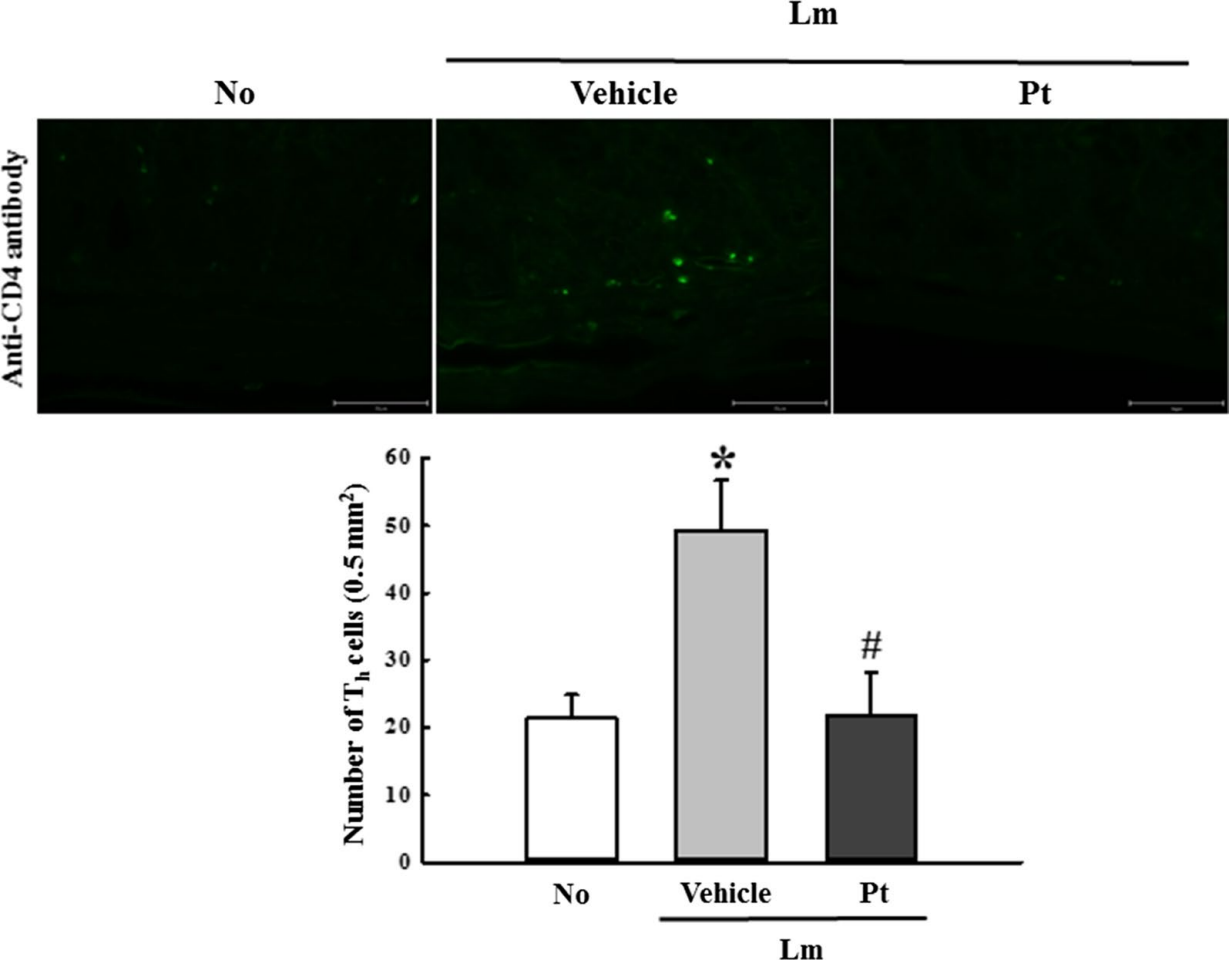
Tissue distribution of CD4^+^ cells. In the mid colon section, the tissue distributions of CD4^+^ cells were analyzed using a specific antibody conjugated with green fluorescence. The slide sections of mid colon tissue were prepared from four to six rats per group, and an immunofluorescence (IF) staining for each sample was analyzed in duplicate. All values in results are represented as the means ± standard deviation (SD). *Indicated statically significance compared to the No treated SD rats, while # indicated statically significance compared to the Lm + Vehicle treated SD rats. Abbreviation: Lm, Loperamide; Pt, Phlorotannins

Finally, the expression level of the inflammatory cytokines in the mid colon was examined because the function of inflammatory mediators was characterized in lamina propria [9]. To investigate if the laxative activity of Pt are accompanied by a recovery of the inflammatory response, the changes in the expression level of TNF-α, IL-6, IL-1β, IL-13, and IL-4 were analyzed in the mid colons of Lm + Pt treated SD rats. The mRNA levels of five cytokines were lower in the Lm + Vehicle treated SD rats than in the No treated SD rats, even though the decrease rate was varied. However, they were recovered after the Pt treatment (Fig. 5). Also, a similar recovery was detected on the distribution of T_h_ cells in the mid colon of Lm + Pt treated SD rats (Fig. 6). These results show that the laxative activity of Pt are linked to the recovery of inflammatory cytokines on the IEB in SD rats with Lm-induced constipation phenotypes.

## Discussion

The IEB is considered one of the largest contact surfaces between the environmental lumen and tissue practices of the body. It is vital for transporting pathogens, nutrients, and water to maintain homeostasis [21, 22]. Hence, histological and molecular changes in the IEB under various pathological conditions of digestive diseases have attracted considerable attention [23]. As part of the above studies, the present study investigated the histological and molecular changes to the IEB in Lm-induced constipation rats during the laxative effects of Pt. The laxative activity of Pt may be associated tightly to improving the morphology and molecular changes of IEB.

The IEB has two key pathways, including the transcellular pathway and paracellular pathway, to pass across solutes and ions from the gut lumen to particular tissue [23]. Among them, the paracellular pathway through the intercellular space is tightly linked to three junctional complexes, including TJ, AJ, and desmosome [24]. In particular, the levels of several transmembrane and cytoplasmic plaque proteins constituting these complexes were changed significantly in IBD and IBS. The expression levels of claudin-2 and 18 for the transmembrane proteins were significantly higher in the patients and animal models with IBD, while these of occludin and ZO-1 for cytoplasmic plaque proteins were lower in the same group [13, 25–27]. A similar decrease pattern in the level of occludin and ZO-1 expression was detected in IBS patients and animal models, but the levels of transmembrane proteins were not analyzed [17, 18, 28]. The present study examined the changes in the expression of the transmembrane and cytoplasmic plaque proteins for TJ and AJ in the mid colon of SD rats with Lm-induced constipation phenotypes. The findings in above model animals are consistent with previous findings in IBD and IBS animals, but the present study analyzed more genes. Furthermore, this study provides the first evidence that constipation may be closely related to regulating the critical factors for TJ and AJ.

Finally, the dysfunction of IEB is link to several intestinal and non-intestinal diseases such as IBD, IBS, intestinal infection, alcoholic liver disease, type I diabetes and emotional stress [29]. During the course of these diseases, the perturbation of IEB was considered to be the cause of body fluid loss, transepithelial migration of neutrophils and bacteria translocation [30]. Also, this dysfunction promotes abnormal delivery of luminal antigen and infiltration of inflammatory cells as well as amplifies the immune response of host. Subsequentially, they lead to the chronicity of IEB dysfunction and enhancing severity of each disease through inducing the over recruitment of neutrophils and secretion of inflammatory cytokines [30, 31]. Furthermore, the paracellular permeability were increased by proinflammatory cytokines, pathogens and several environmental condition through opening tight junctions [30]. In the present study, we examined the expression level of the inflammatory cytokines because an IEB dysfunction is associated with alternative regulation of proinflammatory cytokines [32]. As shown Fig. 5, five cytokines, including TNF-α, IL-6, IL-1β, IL-13, and IL-4, were suppressed in the mid colon of SD rats Lm-induced constipation phenotypes during the dysfunction of the IEB although their level were increased after Pt treatment. In IBD patients, the paracellular permeability in IEB was increased significantly through alternative regulation of the TJ or AJ proteins [3]. In particular, pore-forming claudin-2 expression was upregulated in this disease, whereas claudin-4, 5, 7, and 8 were downregulated in crohn's disease (CD) and ulcerative colitis (UC) [13, 33, 34]. An increased number of pores in IEB lead to the transport of inflammatory infiltrates, including cytokines and other mediators [32]. Some inflammatory cytokines, such as TNF-α, IL-4, IL-13, IFN-γ, IL-1β, IL-9, and IL-16, were implicated in the increase in intestinal permeability during this process [35–38]. Among them, the levels of TNF-α and IFN-γ were higher in the mucosa of IBD patients with IEB disruption [39, 40]. In addition, the expression levels of various inflammatory cytokines were similarly increased in the mid colon of constipation model induced by microplastics treatment although IEB dysfunction has not been examined [41]. The differences between the present study results and previous studies were attributed to some differences in the induction method and molecular mechanism of the disease. Further studies will be needed to address this issue.

## Conclusions

This study examined whether the improvement of the IEB plays a role in the laxative activity of Pt in SD rats with Lm-induced constipation phenotypes. The results suggest that the laxative activity of Pt may be accompanied by the histological recovery of IEB and the alternative expression of junctional complex and inflammatory cytokines in the mid colon of SD rats with Lm-induced constipation phenotypes. Therefore, the histological and molecular changes in the IEB can be considered an important target for treating constipation, but additional research will be needed to verify the action mechanisms.

## Methods

### Preparation of Pt

Pt was prepared using the method previously described in several studies [20, 42]. After mixing the dried *E. cava* powder (30 g) and 70% ethanol (300 mL; v/v), the extract solution was collected by shaking for 12 h at 37°C. The extract solution obtained over three times were filtered and then evaporated at 40°C. These extracts were dissolved in dH_2_O, and sequentially fractionated using three different solvent including n-hexane, chloroform, and ethyl acetate. Finally, the fraction of ethyl acetate was evaporated at 40°C to remove the solution. This pellet was stored as a Pt sample at − 20°C until needed.

### Animal study for the therapeutic effects of Pt

The protocol for experimental animal study was carefully reviewed and approved the Pusan National University-Institutional Animal Care and Use Committee (PNU-IACUC) (Approval Number PNU-2019-2458). Eight-week old SD rats (male, 260–280 g) were provided from Samtako BioKorea Inc. (Osan, Korea), and breed in the barrier facility of the PNU-Laboratory Animal Resources Center (PNU-LARC), accredited by the AAALAC International (Unit Number; 001525) and the Korea Food and Drug Administration (KFDA) (Unit Number; 000231). They were provided, ad libitum, with a filtered tap water and a standard irradiated chow diet (Samtako BioKorea Co.). All SD rats were maintained in a specific pathogen-free (SPF) state, strict regulation of light cycle, constant temperature (22 ± 2°C) and relative humidity (50 ± 10%).

The laxative activity of Pt were analyzed as described in previous studies [43, 44]. Briefly, SD rats (n = 21) were allocated to one of two groups; a normal group (No group, n = 10) and a constipation phenotypes group (Lm treated group, n = 20). All rats of Lm treated group were subcutaneously injected with Lm (4 mg/kg weight, Sigma–Aldrich Co., St. Louis, MO, USA) in 0.5% Tween 20 solution twice daily during three days, while vehicle solution was treated to No group. After one day of Lm injection, these rats were further assigned two groups; a Lm + Vehicle treated SD rats (n = 7) and Lm + Pt treated SD rats (n = 7). A single dose of Pt (50 mg/kg body weight) in 1 × PBS solution orally administered to rats of Lm + Pt treated SD rats, while the vehicle solution administered to Lm + Vehicle treated SD rats under the same pattern. At the same time on the 5^th^ day, the total feces, urine, remaining water, and remaining foods were harvested from the metabolic cage breed with each rat, and alteration on their levels were determined. Finally, all SD rats used in the present study were then euthanized using CO_2_ gas in accordance with the guidelines, after which tissue samples of mid colon were collected and stored at − 70 °C until further analyses.

### Measurement of feces related parameters and urine volume

For the experimental period of time, all SD rats of subset group were individually maintained in metabolic cages to harvest feces and urine samples without contamination. To analysis feces parameters, all feces were collected from each SD rat at the 5th day, and their weights were measured in duplicate using an electric balance (Mettler Toledo S.A.E., Barcelona, España). Also, total number of stools was counted twice per SD rat of subset group, and their morphological image was taken with a digital camera. The water content of feces was further analyzed as follows:

$${\text{Water}}\;{\text{content}}\;{\text{of}}\;{\text{stools}} = \left( {{\text{A}} - {\text{B}}} \right)/{\text{A}} \times {1}00$$

where A is total weight of fresh feces, and B is total weight of feces dried at 60°C for 24 h. Moreover, total volume of urine collected at the 5^th^ day was analyzed using a mass cylinder.

### Measurement for transit rate and length of GI tract

The transit rate and length of total GI tract were measured using the method described elsewhere [20]. Briefly, SD rats fasted for 12 h were administered 0.3 mL of a charcoal meal solution (3% suspension of activated charcoal in 0.5% aqueous methylcellulose) (Sigma–Aldrich Co.). At 30 min after administration of charcoal meal, all rats were euthanized with CO_2_, and the GI tract length from the stomach to the anus and transit distance of charcoal meal was measured in duplicate using a ruler. Finally, the transit rate was determined using the following calculation method:

$$\begin{aligned} {\text{GI}}\;{\text{transit}}\;{\text{ratio}}\left( \% \right) & = \left[ {\left( {{\text{length}}\;{\text{of}}\;{\text{total}}\;{\text{GI}}\;{\text{tract}}{-}{\text{transit}}\;{\text{distance}}\;{\text{of}}\;{\text{charcoal}}\;{\text{meal}}} \right)} \right. \\ & \quad \left. {\left. {/{\text{length}}\;{\text{of}}\;{\text{total}}\;{\text{GI}}\;{\text{tract}}} \right)} \right] \times {1}00 \\ \end{aligned}$$

### Western blotting analysis

The total tissue proteins were prepared from the mid colons of SD rats, using the Pro-Prep Protein Extraction Solution (Intron Biotechnology Inc., Seongnam, Korea). After homogenizing tissues of mid colon, total proteins were harvested, and their concentration were sequentially determined using a SMARTTM Bicinchoninic Acid Protein assay kit (Thermo Fisher Scientific Inc., Waltham, MA, USA). And then, proteins bounded on the nitrocellulose membranes were incubated with the following primary antibodies overnight at 4°C: anti-E-cadherin (24E10, 1:1,000, Cell Signaling Technology Inc., Danvers, MA, USA) or anti-actin (4967 s, 1:3,000, Sigma–Aldrich Co.). The intensity for each protein was analyzed on the membrane, which developed with a Chemiluminescence Reagent (Pfizer Inc., Warren, NJ, USA) using the FluorChem^®^ FC2 imaging system (Alpha Innotech Corporation, San Leandro, CA, USA). Finally, the density of each protein was quantified using the AlphaView Program (Cell Biosciences Inc., Santa Clara, CA, USA).

### Quantitative real time–polymerase chain reaction (qRT-PCR) analysis

The relative quantities of junctional components (p120-catenin, ZO-1, occludin, claudin-1, claudin-4) and inflammatory cytokines (TNF- α, IL-6, IL-1β, IL-13, and IL-4) mRNA were assayed by qRT-qPCR analyses [45, 46]. After isolating total RNA molecules using RNA Bee solution (Tet-Test Inc., Friendswood, TX, USA), complement DNA (cDNA) was synthesized with reverse transcriptase (Superscript II, Thermo Fisher Scientific Inc.). Specific gene was amplified with 2 × Power SYBR Green (Toyobo Co., Osaka, Japan) [47] using specific primers (Additional file 1: Table S1). Finally, the expression of each gene was quantified as relative level to that of the β-actin (housekeeping gene) by comparing the Cts at a constant fluorescence intensity [48].

### Analysis for histopathological structure

The mid colons collected from SD rats were fixed in 10% formalin, and then embedded in paraffin wax. After sectioning into 4 μm thick slices, they were stained using hematoxylin and eosin solution (H&E, Sigma-Aldrich Co.). The histological features on these sections were observed by light microscopy, after which the mucosal layer thickness and muscle thickness for constipation as well as mucus layer, epithelial cells, and lamina propria for IEB abnormality in the mid colon were observed using the Leica Application Suite (Leica Microsystems, Glattbrugg, Switzerland).

### Immunofluorescence (IF) staining analysis

The tissue distribution of T_h_ cells was analyzed with IF staining analysis using anti-CD4 antibody. After deparaffinization of tissue sections, anti-CD4 primary antibody (1:100, BioLegend, San Diego, California, USA) and goat fluorescein isothiocyanate (FITC)- labeled anti-rabbit IgG (1:200, Thermo Fisher Scientific Inc.) were sequentially treated onto these section. Finally, green fluorescence intensity for CD-4 proteins was analyzed using an EVOS M5000 Imaging System (Thermo Fisher Scientific Inc.).

### Statistical analysis

One-way ANOVA used to determine the statistical significance between Lm + Vehicle treated group and Lm + Pt treated group, and only *p* value less than 0.05 was reported as statistically significant. All values in results are represented as the means ± standard deviation (SD).

## Supplementary Information

**Additional file 1.** **Supplement Table S1**. Primer sequences for RT-PCR.

## Data Availability

The datasets used and analyzed during the current study are available from the corresponding author on reasonable request.

## Abbreviations

AAALAC: Association for Assessment and Accreditation of Laboratory Animal Care
AJ: Adherens junction
cDNA: Complement DNA
DNBS: Dinitrobenzene sulfonic acid
DSS: Dextran sodium sulfate
GI: Gastrointestinal
H&E: Hematoxylin and eosin
IBD: Inflammatory bowel disease
IBS: Irritable bowel syndrome
IEB: Intestinal epithelial barrier
KFDA: Korea Food and Drug Administration
Lm: Loperamide
Pt: Phlorotannin
qRT-PCR: Quantitative real-time–polymerase chain reaction
SD: Sprague Dawley
SDS-PAGE: Sodium dodecyl sulfate–polyacrylamide gel electrophoresis
TJ: Tight junction
