# Supplementary material for: Improvement of the intestinal epithelial barrier during laxative effects of phlorotannin in loperamide-induced constipation of SD rats
Source: Lab Anim Res. 2023 Jan 3;39:1. doi: 10.1186/s42826-022-00152-1 (PMC9808941; doi:10.1186/s42826-022-00152-1)
Supplement: Supplementary file 1 — Additional file 1. Supplement Table S1. Primer sequences for RT-PCR. [file 42826_2022_152_MOESM1_ESM.docx]

**Supplement Table S1. Primer sequences for RT-PCR**

| Primer name | Sequence (from 5’ to 3’) | Product size (bp) |
| --- | --- | --- |
| p120-catenin  Forward  Reverse | TGGAC CATGC GCTAC ACGCC  CCGAA GTTTC CGCCG GGCTT | 179 |
| ZO-1  Forward  Reverse | CCTCC GTTGC CCTCA CAGTA  GGGCG CCCTT GGAAT G | 79 |
| Occludin  Forward  Reverse | TTGAA GAGTG GGTTA AAAAT GTGTC T  TCAAC TCTTT CCGCA TAGTC AGAT | 79 |
| Claudin-1  Forward  Reverse | CCCCG GAAAA CAACC TCTTA C  TGTCA CACAT AGTCT TTCCC ACTAG AA | 80 |
| Claudin-4  Forward  Reverse | CGTGG CAAGC ATGCT GATTA  GTCGC GGATG ACGTT GTG | 61 |
| TNF-α  Forward  Reverse | ATCCG CGACG TGGAA CTG  ACCGC CTGGA GTTCT GGAA | 70 |
| IL-6  Forward  Reverse | TTGGG ACTGA TGTTG TTGAC A  TCATC GCTGT TGATA CAATC AGA | 200 |
| IL-1β  Forward  Reverse | CTACA GGCTC CGAGA TGAAC AAC  TCCAT TGAGG TGGAG AGCTT TC | 79 |
| IL-13  Forward  Reverse | CCTTA AGGAG CTTAT TGAGG AGCTG AG  CAGTT GCTTT GTGTA GCTGA GCAG | 280 |
| IL-4  Forward  Reverse | GAATA TACCA GGAGC CATAT C  CTCAG TACTA CGAGT AATCC A | 385 |
| β-actin  Forward  Reverse | TGG AAT CCT GTG GCA TCC ATG AAA C  TAA AAC GCA GCT CAG TAA CAG TCC G | 349 |
